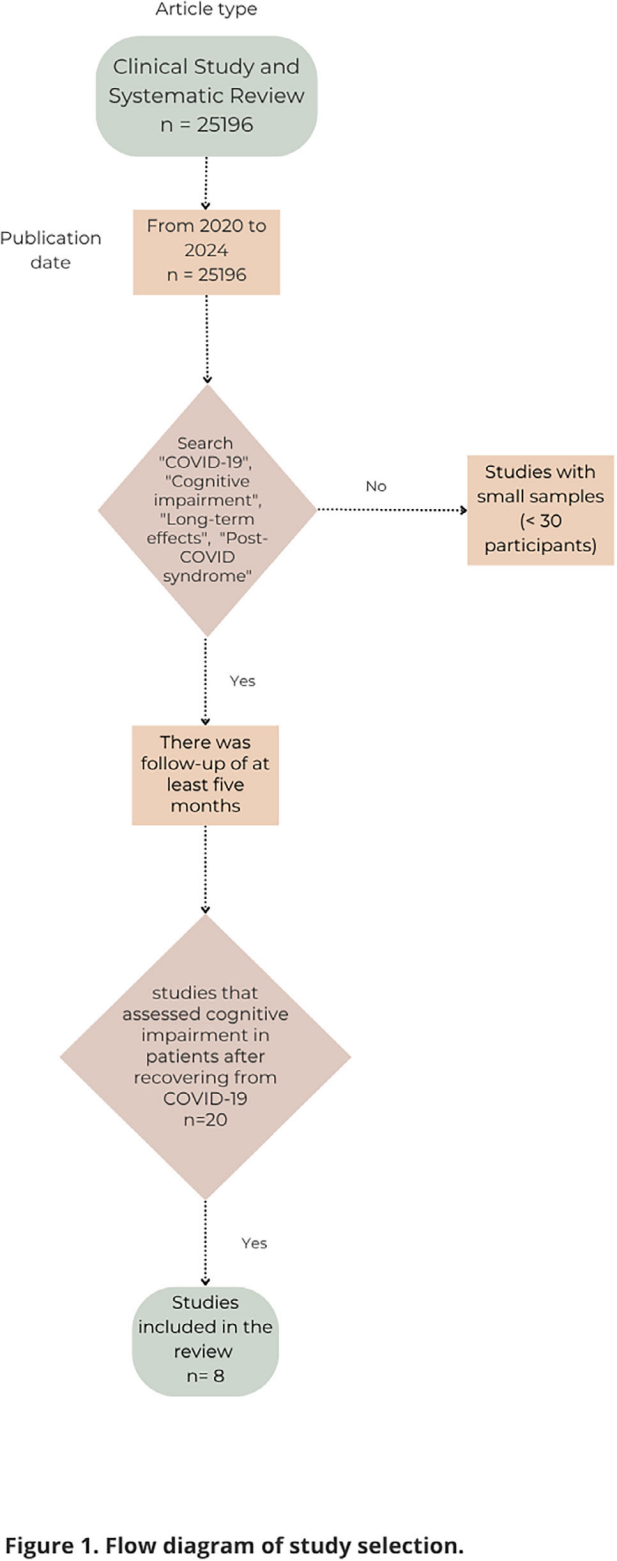# Long‐term Cognitive Impairment Following 2019 Covid Pandemic: A Scoping Review

**DOI:** 10.1002/alz70857_105305

**Published:** 2025-12-25

**Authors:** Camila Rafaela Lazaretti, Cristiano Aguzzoli

**Affiliations:** ^1^ Feevale University, Novo Hamburgo, RS, Brazil

## Abstract

**Background:**

The COVID‐19 pandemic significantly impacted the overall health of those affected, often harming general cognition distinct and higher cortical functions. Recent studies suggest that the infection may result in prolonged cognitive deficits. This study aims to review differences in long‐term cognitive impairment in individuals who developed mild‐to‐moderate COVID‐19 symptoms relative to severe cases.

**Method:**

We conducted a scoping review on the long‐term cognitive effects of COVID‐19, selecting articles from 2020 to 2024 indexed in the PubMed database. Inclusion criteria required studies that assessed long‐term cognitive impairment in individuals recovering from COVID‐19.

**Result:**

A total of twenty articles were screened for eligibility, and eight full‐text publications were assessed (Figure 1). The prevalence of cognitive impairment in the acute phase of COVID‐19 ranged from 61.5% in mild to moderate cases to 80% in moderate to severe cases. We found that executive function, working memory, verbal fluency, and attention were the cognitive domains most impacted. In the long‐term, more than 50% of patients continued to experience cognitive deficits even after full recovery, particularly in attention, memory, and executive function. A Swedish study reported that 48% of severe‐symptom survivors exhibited cognitive deficits—such as short memory deficit—five months after hospital discharge, while another study showed that 38% of moderate and 11.2% of severe cases experienced immediate verbal memory impairment. Another single‐center study assessing 726 survivors of COVID‐19 from the Intensive Care Unit with a median age of 62 years found that 87.5% had not fully regained their daily activity levels, and only 6.2% had returned to their previous functional level.

**Conclusion:**

A growing body of evidence indicates that post‐COVID‐19 cognitive impairment may persist in a significant proportion of survivors, indicating that cognitive impairment are not limited to severe cases but can affect individuals across clinical stages. Among the various cognitive difficulties observed, memory impairment stands out as the predominant domain among patients recovering from COVID‐19. Therefore, the clinical evaluation of long‐term follow‐up of these patients is important, with special attention to cognitive function.